# Systemic Inflammation and Lung Function Impairment in Morbidly Obese Subjects with the Metabolic Syndrome

**DOI:** 10.1155/2013/131349

**Published:** 2013-02-24

**Authors:** Astrid van Huisstede, Manuel Castro Cabezas, Erwin Birnie, Gert-Jan M. van de Geijn, Arjan Rudolphus, Guido Mannaerts, Tjin L. Njo, Pieter S. Hiemstra, Gert-Jan Braunstahl

**Affiliations:** ^1^Department of Pulmonology, Sint Franciscus Gasthuis, Kleiweg 500, 3045 PM Rotterdam, The Netherlands; ^2^Department of Internal Medicine, Sint Franciscus Gasthuis, 3045 PM Rotterdam, The Netherlands; ^3^Department of Statistics, Sint Franciscus Gasthuis, 3045 PM Rotterdam, The Netherlands; ^4^Institute of Health Policy and Management, Erasmus University Rotterdam, 3000 DR Rotterdam, The Netherlands; ^5^Department of Clinical Chemistry, Sint Franciscus Gasthuis, 3045 PM Rotterdam, The Netherlands; ^6^Department of Surgery, Sint Franciscus Gasthuis, 3045 PM Rotterdam, The Netherlands; ^7^Department of Pulmonology, Leiden University Medical Center, 2300 RC Leiden, The Netherlands

## Abstract

*Background*. Obesity and asthma are associated. There is a relationship between lung function impairment and the metabolic syndrome. Whether this relationship also exists in the morbidly obese patients is still unknown. *Hypothesis*. Low-grade systemic inflammation associated with the metabolic syndrome causes inflammation in the lungs and, hence, lung function impairment. *Methods*. This is cross-sectional study of morbidly obese patients undergoing preoperative screening for bariatric surgery. Metabolic syndrome was assessed according to the revised NCEP-ATP III criteria. *Results*. A total of 452 patients were included. Patients with the metabolic syndrome (*n* = 293) had significantly higher blood monocyte (mean 5.3 versus 4.9, *P* = 0.044) and eosinophil percentages (median 1.0 versus 0.8, *P* = 0.002), while the total leukocyte count did not differ between the groups. The FEV_1_/FVC ratio was significantly lower in patients with the metabolic syndrome (76.7% versus 78.2%, *P* = 0.032). Blood eosinophils were associated with FEV_1_/FVC ratio (adj. B −0.113, *P* = 0.018). *Conclusion*. Although the difference in FEV_1_/FVC ratio between the groups is relatively small, in this cross-sectional study, and its clinical relevance may be limited, these data indicate that the presence of the metabolic syndrome may influence lung function impairment, through the induction of relative eosinophilia.

## 1. Introduction

Obesity is an increasing worldwide problem that has taken on epidemic proportions [1]. Cross-sectional studies have shown a positive association between obesity and asthma [2]. Weight loss in obese asthma patients improved morbidity and lung function [3]; however, the mechanisms underlying the relationship between asthma and obesity are unclear. It is suggested that low-grade systemic inflammation associated with obesity plays a role.

The metabolic syndrome is a common metabolic disorder that may result from the increasing prevalence of obesity. Metabolic syndrome is a cluster of cardiometabolic risk factors characterized by abdominal obesity, insulin resistance, and chronic systemic inflammation [4]. Positive associations with lung function impairment have been reported for components of the metabolic syndrome, such as hypertension [5], type diabetes mellitus [6, 7], low-density lipoprotein cholesterol [8], and overall obesity [9]. In recent large cohort studies, it has been shown that there is also a relationship between lung function impairment and the metabolic syndrome [10–12]. However, all the aforementioned studies included overweight as well as normal weight subjects and were therefore not specifically targeted to examine these issues in the morbidly obese. Data on the association between lung function impairment and the metabolic syndrome in the morbidly obese are limited.

The mechanisms underlying the relationship between impaired lung function and the metabolic syndrome are unclear. The chronic low-grade systemic inflammation that is associated with obesity might explain this relationship. Our hypothesis is that this low-grade systemic inflammation causes inflammation in the lungs and, hence, lung function impairment. 

We therefore performed a study to investigate (1) the association between lung function and the metabolic syndrome in morbidly obese subjects and (2) to determine the effect of systemic inflammation on the relationship between lung function impairment and the metabolic syndrome. 

## 2. Methods

### 2.1. Study Population

The subjects included in this study were consecutive patients who underwent preoperative screening for bariatric surgery in the Sint Franciscus Gasthuis in Rotterdam, The Netherlands, between October 2009 and May 2011. Eligibility criteria for bariatric surgery were age between 18 and 60 years and body mass index (BMI) either ≥40 kg/m^2^ or ≥35 kg/m^2^ combined with the presence of comorbidity, for example, diabetes mellitus, hypertension, or proven obstructive sleep apnea syndrome (OSAS). Subjects underwent baseline physical examinations that included routine assessment of anthropometry, blood pressure, pulmonary function, and blood samples. 

Height and weight were measured wearing light clothes and no shoes. Body mass index was calculated as weight (in kg) divided by height (in m) squared. Abdominal circumference was measured directly to the body surface midway between the lower rib margin and the ileac crest. 

The Epworth Sleepiness Scale questionnaire [13] was used to assess OSAS and the GERD questionnaire [14] for gastro-esophageal reflux disease (GERD). 

All subjects gave informed consent, and the local ethics committee (Toetsingscommissie Wetenschappelijk Onderzoek Rotterdam e.o, Trial no. NL25637.101.08) approved the study protocol (The Netherlands Trial Register no. NTR3204). 

### 2.2. Definition of the Metabolic Syndrome

Metabolic syndrome was diagnosed according to the National Cholesterol Education Program's Adult Treatment Panel III report (NCEP ATP-III) criteria when ≥3 of the following 5 risk factors were present: abdominal obesity, an elevated level of serum triglycerides, low serum level of high-density lipoprotein cholesterol (HDL-C), elevated blood pressure, and high serum glucose level or treatment for any of these disorders [4]. 

### 2.3. Pulmonary Function Tests

Spirometry was performed with Vmax spirometer (Vmax SensorMedics Viasys, type Encore 20/22/229/62 Encore, Cardinal Health, USA) before and after 400 *μ*g of inhaled salbutamol, with subjects in a sitting position and nose clips in place according to the American Thoracic Society/European Respiratory Society statement [15]. All values obtained were related to height, age, and gender and expressed as percentage of their predicted value (reference ERS 1993). The pulmonary function results are prebronchodilator values unless specifically noted. Exhaled nitric oxide (FeNO) was measured with NIOX MINO (Aerocrine, Sweden) and expressed in parts per billion (ppb). 

### 2.4. Clinical Chemistry

Blood was taken by venipuncture during routine preoperative screening. Laboratory measurements were performed according to standard procedures by our Department of Clinical Chemistry. Plasma-cholesterol, HDL-cholesterol, glucose, triglycerides, and CRP were measured using LX 20 and DxC analyzers (Beckman Coulter, Miami, FL, USA). LDL-cholesterol was calculated using the Friedewald formula. Total IgE and specific plasma IgE were determined with a solid-phase two-step chemiluminescent immunoassay on the Immulite 2000 (Siemens, Los Angeles, CA). A positive inhalation screen was defined as at least one increased amount of specific IgE against one of the following allergens: *Aspergillus fumigatus*, house-dust mite, cat, dog, grass, birch, or herbs. Blood cell counts and 5-part leukocyte differentiation were determined automatically using LH750 analyzers (Beckman Coulter). HbA1C was determined using a Tosoh G8 HLC-723 analyzer (Tosoh Bioscience, Tokyo, Japan). Insulin was measured using radio immunoassay (RIA) DSL1600 (Diagnostic Systems Laboratories, Webster, TX, USA). Vitamin D was determined by RIA or chemiluminescence (LIA) on Liason analyzers (DiaSorin, Stillwater, MN, USA).

### 2.5. Statistical Analyses

Unadjusted between-group comparisons were performed using Student's *t* test or the Chi-square test, where appropriate, and Mann-Whitney *U* test for nonparametric comparisons (eosinophils). CRP, IgE, insulin, lipoprotein-a, and vitamin B6 were not normally distributed (standard error of kurtosis and skewness below −3 or above 3) and were therefore log transformed. Univariate linear regression was used to evaluate associations between continuous variables. Backward linear regression analysis was used to investigate which component of the metabolic syndrome is related to the FEV_1_/FVC ratio. Variables associated with FEV_1_/FVC ratio in univariate analysis at a *P*-value of <0.1 were examined in the multiple linear regression analysis. Since the FEV_1_/FVC ratio as percentage predicted is already corrected for height, age, and gender, we did not add these variables to the model. As eosinophils and abdominal circumference were correlated, we selected only eosinophils to prevent collinearity. We omitted OSAS because of the large proportion of missing data. All analyses were performed using SPSS 18.0 software (SPSS Inc., Chicago, IL, USA). Results were evaluated at 95% confidence interval at a two-sided *P* < 0.05. 

## 3. Results

### 3.1. Subjects

A total of 452 subjects were included in the study (97 males and 355 females). Also 293 subjects (64.8%) fulfilled the criteria for metabolic syndrome.  Table 1 shows the general characteristics of the study population. Patients with the metabolic syndrome were significantly older (mean 43 years), were less often female (73.7%), and had a larger abdominal circumference in centimeters (mean 134.3 cm) compared to patients without the metabolic syndrome (mean 37 years, 87.4%, and mean 126.8 cm, resp.). 

### 3.2. Metabolic Syndrome and Blood Parameters

Patients with the metabolic syndrome did not have a higher total leukocyte count or neutrophil percentage in the peripheral blood (*P* = 0.253), but they did have a significantly higher percentage of eosinophils (*P* = 0.002) and monocytes (*P* = 0.044) compared to patients without the metabolic syndrome (Table 2). Other parameters of systemic inflammation such as CRP, Complement C3, and Complement C4 did not show significant differences between the two groups (Table 2). There was no correlation between BMI and eosinophils. We did find a low, but significant, correlation between eosinophils and abdominal circumference (Spearman correlation coefficient 0.270, *P* < 0.001) and CRP and abdominal circumference (Spearman correlation coefficient 0.146, *P* = 0.010). 

### 3.3. Metabolic Syndrome and Pulmonary Function Tests

The subjects with the metabolic syndrome showed a significantly lower FEV_1_/FVC ratio—a measure for bronchial obstruction—compared to the group without the metabolic syndrome (76.7% and 78.2%, resp., *P* = 0.032), while no difference was observed regarding FEV_1_ or FVC (Table 3). Although there was no significant difference in FEF_25–75_ between subjects with and without the metabolic syndrome, the FEF_75_ was significantly lower in the metabolic syndrome group (*P* = 0.036). Log transformed FeNO—a measure for bronchial inflammation—showed no difference between the two groups. There was no correlation between BMI and FeNO and abdominal circumference and FeNO. 

### 3.4. Subgroup Analysis: Self-Reported Asthma

There was no difference in the prevalence of self-reported asthma between subjects with or without the metabolic syndrome (21.0% versus 19.6%, resp., *P* = 0.723). In a subgroup analysis of subjects with self-reported asthma, we found no significant difference in the use of inhaled corticosteroids, body mass index, pack years, FEV_1_ (% predicted), FEV_1_/FVC (% predicted), FeNO, blood eosinophils, or CRP between subjects with or without the metabolic syndrome (data not shown). 

### 3.5. Subgroup Analysis: Reversibility

In a subgroup analysis of subjects with and without reversibility (ΔFEV_1_ ≥ 12%), we found that only 50% of subjects with reversibility (*n* = 30) had self-reported asthma. Although subjects with reversibility did use more often inhaled corticosteroids (13.3% versus 4.4%, *P* = 0.032), there was no significant difference in FeNO, blood eosinophils, or CRP between subjects with and without the metabolic syndrome.

### 3.6. Variables Related with FEV_1_/FVC Ratio

Since the FEV_1_/FVC ratio was the only variable of spirometric function tests which was different between subjects with and without the metabolic syndrome and our hypothesis was that the metabolic syndrome causes a lower FEV_1_/FVC ratio—not the other way around—we investigated which variables were related to the FEV_1_/FVC ratio. 

There was an association between the peripheral blood eosinophils percentage and the FEV_1_/FVC ratio (univariate linear regression coefficient = −0.806, *P* = 0.006). Abdominal circumference and OSAS (Epworth Sleepiness Scale (ESS)) were significantly related to the FEV_1_/FVC ratio (univariate linear regression analysis), whereas BMI, GERD, CRP and monocytes were not significantly related to the FEV_1_/FVC ratio (Table 4). Missing values of ESS were not correlated to other variables.

Since the metabolic syndrome was associated with the FEV_1_/FVC ratio, we investigated which of the components of the metabolic syndrome contributed to this relationship. Only hypertension was significantly related to FEV_1_/FVC ratio (regression coefficient = −1.612, *P* = 0.038 in backward linear regression analysis) (Table 5). 

### 3.7. Multiple Regression Analysis

After correction for the use of inhaled corticosteroids and the number of pack years, we found an association between blood eosinophils and the FEV_1_/FVC ratio (adj. B −0.113, *P* = 0.018) (Table 6). 

## 4. Discussion

This study shows that obese patients with the metabolic syndrome have a higher proportion of blood monocytes and eosinophils and a lower FEV_1_/FVC ratio, indicating airway obstruction, than obese patients without the metabolic syndrome. Blood eosinophils (%) in morbidly obese subjects were related to FEV_1_/FVC, whereas monocytes were not. After adjustment for multiple variables, the relationship between FEV_1_/FVC ratio and eosinophils remained intact. Although the differences are small, it strengthens our hypothesis that the presence of the metabolic syndrome could play a role in lung function impairment, through the induction of systemic inflammation, in particular mediated by blood eosinophils. Whether this also leads to asthma on the long term still remains to be elucidated. 

To our knowledge, this is the first study concerning lung function and the metabolic syndrome in a cohort of only morbidly obese subjects. Secondly, comorbid conditions associated with obesity such as OSAS and GERD were taken into account in the current study. Thirdly, adiposity was not only assessed with BMI, but also with abdominal circumference. Although one would expect that all our subjects have the metabolic syndrome, we found a 65% prevalence of the metabolic syndrome in our group, which corresponds with 60% of the morbidly obese in the NHANES III cohort [16].

Various studies have shown that obesity causes a modest reduction in total lung capacity (TLC) and a larger reduction in functional residual capacity (FRC) [17]. However, we were unable to perform a TLC measurement in all subjects to rule out a restrictive pattern. The FEV_1_/FVC ratio is usually well preserved in obese subjects, or even increased. This could explain the high mean FEV_1_/FVC ratio in our subjects, and therefore we have used the FEV_1_/FVC ratio as a continuous variable and did not use a cutoff value of 70% predicted. Furthermore, in contrast to the FEV_1_/FVC, the FEV_1_ is influenced by BMI; hence, our focus is on the FEV_1_/FVC ratio. 

The prevalence of self-reported asthma was similar in the two groups. We did not perform methacholine-provocation tests; so, a definitive diagnosis of asthma was often not possible. Since misdiagnosis of asthma is a relevant issue, also in the obese [18], we felt more comfortable using objective parameters instead of a presumed diagnosis. 

Since the metabolic syndrome was associated with the FEV_1_/FVC ratio in univariate analysis, we investigated which of the components of the metabolic syndrome contributed to this relationship. Hypertension was the only component that was significantly associated with a reduced FEV_1_/FVC ratio after multiple backward regression analysis. Hypertension may have the strongest association with systemic inflammation, as proposed by Irace et al. [19]. We could also confirm the results of Leone et al. [10] showing that there is indeed a relationship between abdominal circumference (in cm) and the FEV_1_/FVC ratio. Interestingly, we found a correlation between blood eosinophil percentage and abdominal circumference, where we found no correlation between blood eosinophil percentage and BMI, indicating that it is mainly the place of the fat that matters. This suggests that the increased abdominal circumference of subjects with the metabolic syndrome causes the relative eosinophilia.

Obesity is a state of chronic low-grade systemic inflammation. Leukocyte count is considered a marker of systemic inflammation. Several epidemiological studies have already noted a relationship between some components of metabolic syndrome and leukocytes [20, 21]. Several studies showed an increased eosinophil percentage in obesity [22] or in the metabolic syndrome [20, 21]. C-reactive protein (CRP) is a traditional marker of inflammation and is well correlated with BMI [23]. Even though our study found no difference in CRP between the two groups—we did not use high-sensitivity CRP measurements—monocyte and eosinophils percentage in the blood were higher in those with the metabolic syndrome. Our study suggests that increased numbers or circulating eosinophils could be a specific manifestation of the systemic inflammation associated with the metabolic syndrome.

The question remains why there is relative eosinophilia in the obese. Traditionally, eosinophils are related to allergic diseases, asthma, and parasitic infections. The fat tissue is a source of adipokines, which are considered to play a role in the low-grade systemic inflammation in obesity [24]. Leptin—mainly produced by adipose tissue—is a proinflammatory agent. Serum leptin is markedly increased in obese humans, correlating to BMI [25]; it activates eosinophils [26] and increases their survival [27]. Serum leptin levels are elevated in adults of normal weight with impaired lung function [28]. Eotaxin is an eosinophil-specific chemokine that is increased in obesity [29]. Adipocytes produce more eotaxin when stimulated by leptin [30]. Plasma adiponectin—an anti-inflammatory hormone—is reversely correlated to BMI [31]. The leptin/adiponectin ratio is a marker of insulin resistance [32] and the metabolic syndrome [33]. Thus, both leptin and possibly eotaxin could contribute to relative eosinophilia in the obese. Unfortunately, we do not have data on leptin, adiponectin, and eotaxin to further support their role in the observed eosinophilia. 

Another question is whether this relative eosinophilia in the peripheral blood also has local effects on the bronchial tissue and causes or enhances the bronchial inflammation as seen in asthma. Asthma-like symptoms are common in patients with the metabolic syndrome, and their pulmonary function is impaired [34, 35]. Although we did find differences in pulmonary function between subjects with and without the metabolic syndrome, there was no difference in prevalence of self-reported asthma between the two groups. This is, however, unreliable in the morbidly obese [18]. The characteristics of airway inflammation in asthma (exhaled nitric oxide (eNO) and inflammatory cells in induced sputum) have been investigated in obesity and are still subject to debate. de Winter-de Groot et al. showed that a higher BMI was associated with a higher eNO in healthy patients [36]. Several studies have found no—or even an inverse—relationship between the number of sputum eosinophils and BMI [37]. We found no difference in eNO between subjects with and without the metabolic syndrome. Induced sputum or bronchial biopsies could have helped to solve this question but were unfeasible in this study. Since there was no difference in the use of inhaled corticosteroids, this could not have influenced our results of eNO.

Our study includes several limitations. We did not measure TLC values; so, we were not able to firmly assess a restrictive lung function pattern. Also, we did not perform methacholine-provocation tests; so, a definite diagnosis of asthma was often not possible. The FEV_1_/FVC ratio, however, is easy to measure and widely used in clinical practice. Although the differences in FEV_1_/FVC and eosinophils between subjects with and without the metabolic syndrome were small, the difference was significant despite the fact that our study groups consisted of unselected subjects. There probably is a selection bias since we only included patients who were willing to undergo bariatric surgery. It is widely known that most of the subjects who undergo bariatric surgery are females. This explains the female predominance among our subjects. Furthermore, we did not measure adipocytokines or high-sensitivityCRP. We did not include a non-obese control group as comparison for low-grade inflammation, and we cannot fully exclude that smoking might have contributed to a state of low-grade inflammation. However, we corrected for smoking in the multiple regression analysis. Finally, we realize that a cross-sectional association between metabolic syndrome and lung function cannot establish causality. However, Naveed et al. [12] have recently shown that the metabolic syndrome predicts a steeper FEV_1_ decline over time, suggesting that the systemic inflammation produced by metabolic syndrome may impact the progression to abnormal lung function in a longitudinally followed cohort. 

In summary, our study shows that there is a small, but statistically significant, difference in eosinophils and FEV_1_/FVC between subjects with and without the metabolic syndrome. After correction for other variables, an association between blood eosinophils and FEV_1_/FVC remained. Although the differences we have found were relatively small, it might support our hypothesis that the presence of the metabolic syndrome may influence lung function impairment, through the induction of systemic inflammation, in particular, mediated by blood eosinophils. Further research with a firm diagnosis of asthma and assessment of peripheral airway inflammation is necessary. Moreover, in order to establish causality between the metabolic syndrome and lung function impairment, longitudinal studies in morbidly obese patients before and after bariatric surgery are needed.

## Figures and Tables

**Table 1:** General characteristics of the population included in the study.

	No metabolic syndrome (*n* = 159)	Metabolic syndrome (*n* = 293)	*P* value
Age (years)	37 ± 11	43 ± 10	<0.001^1^
BMI (kg/m^2^)	45.4 ± 6.7	46.0 ± 6.5	0.395
Gender (% female)	87.4%	73.7%	0.001^2^
Ethnicity (% Caucasian)	80.5%	85.0%	0.222
Smoking			0.152
% never smoked	49%	40%	
% stopped smoking	29%	32%	
% smokes	22%	28%	
Pack years^3^	6.0 ± 11.2	9.9 ± 14.8	0.003
Abdominal circumference (cm)	126.8 ± 14	134.3 ± 16	<0.001
Systolic blood pressure (mm Hg)	136.4 ± 17.9	145.98 ± 16.6	<0.001
Diastolic blood pressure (mm Hg)	83.6 ± 11.8	87.83 ± 10.1	<0.001
Comorbidities			
Diabetes mellitus	3.8%	30.3%	<0.001
Hypertension	18.9%	42.8%	<0.001
Hypercholesterolemia	1.9%	21.7%	<0.001
Self-reported asthma	19.6%	21.0%	0.723
Use of inhaled corticosteroids	3.8%	5.5%	0.413

^1^Data presented as mean ± standard deviation, *P* value for Student's *t* test,

^2^Data presented as percentage, *P* value for Chi-square test.

^3^Data were log transformed for statistical analysis.

**Table 2:** Comparison of blood parameters between subjects with and without metabolic syndrome.

	No metabolic syndrome (*n* = 159)	Metabolic syndrome (*n* = 293)	*P* value
Leukocytes (10^9^/L)	8.6 ± 2.3	8.9 ± 2.1	0.253^1^
Neutrophils (%)	70.1 ± 8.6	68.9 ± 9.1	0.186
Lymphocytes (%)	23.7 ± 7.1	23.8 ± 7.0	0.844
Monocytes (%)	4.9 ± 1.8	5.3 ± 1.9	0.044
Eosinophils (%)	0.82 (0.05–1.07)	1.00 (0.45–1.85)	0.002^2^
CRP (mg/L)^3^	9.6 ± 7.4	9.5 ± 8.0	0.865
Cholesterol (mmol/L)	5.2 ± 1.0	5.1 ± 1.1	0.198
HDL-cholesterol (mmol/L)	1.3 ± 0.3	1.1 ± 0.2	<0.001
LDL-cholesterol (mmol/L)	3.5 ± 0.9	3.3 ± 1.1	0.108
Triglyceride (mmol/L)	1.0 ± 0.4	1.6 ± 1.0	<0.001
Glucose (mmol/L)	6.3 ± 1.3	8.2 ± 3.9	<0.001
HbA1C (%)	5.52 ± 0.4	6.4 ± 1.5	<0.001
Insulin (mE/L)^3^	56.2 ± 58.1	75.0 ± 79.4	<0.001
Vitamin D (nmol/L)	39.5 ± 22.9	38.5 ± 17.7	0.661
IgE (kU/L)^3^	213.5 ± 479.9	197.8 ± 391.3	0.141
Positive inhalation screen	46.2%	44.0%	0.694

^1^Data presented as mean ± standard deviation, *P* value for Student's *t*-test.

^2^Data presented as median (1st–3rd quartiles), *P* value for Mann-Whitney *U* test.

^3^Data were log transformed for statistical analysis.

**Table 3:** Pulmonary function test of subjects with and without metabolic syndrome.

	No metabolic syndrome (*n* = 159)	Metabolic syndrome (*n* = 293)	*P* value
FEV_1_ (% predicted)	93.0 ± 13.9	91.2 ± 14.5	0.206^3^
FVC (% predicted)	100.6 ± 14.0	98.5 ± 14.7	0.206
FEV_1_/FVC	78.2 ± 6.9	76.7 ± 6.4	0.032
FEF_25–75_ (% predicted)	77.4 ± 24.0	73.9 ± 23.3	0.152
Reversibility FEV_1_ > 12%	6%	7%	0.523^2^
FeNO (ppb)^1^	19.3 ± 22.4	17.6 ± 12.6	0.541

^1^Data were log transformed before comparison.

^2^Data presented as percentage, *P* value for Chi-square analysis.

^3^Data presented as mean ± standard deviation, *P* values for Student *t*-test.

FEV_1_: forced expired volume in 1 second; FVC: forced vital capacity; FEF_25–75_: forced expiratory flow 25%–75%; TLC: total lung capacity; FeNO: fractional expiratory nitric oxide.

**Table 4:** Univariate linear regression analysis of FEV_1_/FVC.

	Regression coefficient	Standard error	*P* value^2^
Monocytes (%)	−0.079	0.162	0.626
Eosinophils (%)	−0.806	0.290	0.006
CRP (mg/mL)	0.075	0.045	0.098
Abdominal circumference (cm)	−0.058	0.021	0.006
Body mass index (kg/m^2^)	0.041	0.047	0.387
OSAS (Epworth Sleepiness Scale)^1^	0.311	0.141	0.029
GERD (GERD questionnaire)	0.068	0.157	0.667

^1^Missing values *n* = 148.

^2^Data presented as linear regression coefficient, *P* value for simple linear regression analysis.

OSAS: obstructive sleep apnea syndrome; GERD: gastro esophageal reflux disease.

**Table 5:** Univariate regression analysis of relation of components of metabolic syndrome and FEV_1_/FVC ratio.

	Regression coefficient	Standard error	*P* value^1^
Abdominal circumference	NA		
Hypertriglycemia	−1.119	0.739	0.130
Low serum HDL-cholesterol	0.712	0.686	0.300
Hypertension	−1.612	0.774	0.038
High serum glucose	−0.010	0.753	0.989

^1^Data presented as linear regression coefficient, *P* value for linear regression analysis.

Abdominal circumference is not applicable, because all patients fulfill the criteria for abdominal circumference according to the NCEP-ATP III criteria for metabolic syndrome.

**Table 6:** Multiple regression analysis FEV_1_/FVC ratio.

	Whole group (*n* = 408) (Adj *R* ^2^ = 0.074)		
	Regression coefficient	95% CI	*P* value
Eosinophils (%)	−0.113	−1.247–−0.118	0.018
Use of inhalation corticosteroids	−0.025	−3.635–2.122	0.606
Pack years	−0.259	−0.162–−0.076	<0.001

Variables associated with FEV_1_/FVC ratio in univariate analysis at a *P* value of <0.1 were examined in the multiple linear regression analysis. Since the FEV_1_/FVC ratio as percentage predicted is already corrected for height, age, and gender, we did not add this variable to the model. As eosinophils and abdominal circumference are correlated, we selected only one variable to prevent colinearity. We omitted OSAS because of the large proportion of missing data.

## Abbreviations

BMI: Body mass index
CRP: C-reactive protein
ESS: Epworth Sleepiness Scale
FEF_25–75_: Forced expiratory flow 25%–75%
FeNO: Fraction of exhaled nitric oxide
FEV_1_: Forced expiratory volume in 1 second
FVC: Forced vital capacity
GERD: Gastroesophageal reflux disease
HDL: High-density lipoprotein
NCEP-APT III: National Cholesterol Education Program's Adult Treatment Panel III report
OSAS: Obstructive sleep apnea syndrome.
